# The Potential of Virtual Reality for the Investigation of Awe

**DOI:** 10.3389/fpsyg.2016.01766

**Published:** 2016-11-09

**Authors:** Alice Chirico, David B. Yaden, Giuseppe Riva, Andrea Gaggioli

**Affiliations:** ^1^Dipartimento di Psicologia, Università Cattolica del Sacro CuoreMilan, Italy; ^2^Department of Psychology, University of PennsylvaniaPhiladelphia, PA, USA; ^3^Applied Technology for Neuro-Psychology Lab, IRCCS Istituto Auxologico ItalianoMilan, Italy

**Keywords:** awe, emotional induction, virtual reality, presence, ecological validity

## Abstract

The emotion of awe is characterized by the perception of vastness and a need for accommodation, which can include a positive and/or negative valence. While a number of studies have successfully manipulated this emotion, the issue of how to elicit particularly intense awe experiences in laboratory settings remains. We suggest that virtual reality (VR) is a particularly effective mood induction tool for eliciting awe. VR provides three key assets for improving awe. First, VR provides users with immersive and ecological yet controlled environments that can elicit a sense of “presence,” the subjective experience of “being there” in a simulated reality. Further, VR can be used to generate complex, vast stimuli, which can target specific theoretical facets of awe. Finally, VR allows for convenient tracking of participants’ behavior and physiological responses, allowing for more integrated assessment of emotional experience. We discussed the potential and challenges of the proposed approach with an emphasis on VR’s capacity to raise the signal of reactions to emotions such as awe in laboratory settings.

## Introduction

When we encounter something greater than ourselves, we can experience “awe.” This emotion, described psychologically by Keltner and Haidt (2003) as “in the *upper reaches of pleasure and on the boundary of fear”* (p. 297), is a response to a perception of vastness that challenges our mental schemas. Awe is a complex and powerful emotion. It can, for example, make us aware of the enormity of existence and our relatively small place in it (Keltner and Haidt, 2003; Saroglou et al., 2008). Despite its profundity, awe has only just begun to receive rigorous empirical attention.

According to Keltner and Haidt (2003), awe contains two key dimensions: vastness and the need for accommodation. Vastness involves the perception of enormous and/or complex stimuli, such as a grand view (perceptual vastness) or a big idea (conceptual vastness). The need for accommodation describes how awe-inducing stimuli force us to adjust our cognitive schemas to accommodate them. This aspect may also imply attentional salience and novelty, therefore, at the cognitive level, the need for accommodation component has been related to surprise (Keltner and Haidt, 2003). Specifically, it could be possible to identify a link between the need for accommodation and specific concepts of surprise (e.g., astonishment), since they can entail the revision of our *well-consolidated beliefs* (Lorini and Castelfranchi, 2007) and because both these emotions belong to the family of *knowledge emotions* (Silvia, 2010). Moreover, awe can have both a positive and/or a negative valence, depending, in part, on how the experience is interpreted (Keltner and Haidt, 2003). Despite this theoretical complexity, most research focus on perceptual vastness and on positive experiences of awe (e.g., Griskevicius et al., 2010; Campos et al., 2013; Prade and Saroglou, 2016), while conceptual vastness, negative experiences of awe, and the need for accommodation have only rarely been addressed (Shiota et al., 2007; Schurtz et al., 2012; Valdesolo and Graham, 2014; Piff et al., 2015). Importantly, while previous experimental studies constructively induced awe in lab setting, they tended to evoke a low intensity version of awe. In other words, awe experiences elicited in laboratory settings have tended to be somewhat subtle (Silvia et al., 2015).

Here, we suggest virtual reality (VR) as an approach to elicit awe in more intense ways in laboratory settings. VR is the combination of stereoscopic displays, real-time motion-tracking, and stereo headphones as well as other possible sensory simulations (e.g., haptic, olfactory) to create simulated environments. Thanks to its ability to generate realistic simulations of real-world experiences, VR has shown effective as mood-induction procedure to elicit different types of emotions (Riva et al., 2007, 2016b; Gorini et al., 2011; Diemer et al., 2015). After reviewing these preliminary experimental studies, we argue that VR provides three key assets for improving awe. First, VR provides users with immersive, realistic, and interactive environments that can elicit a sense of “presence,” that is, the subjective experience of “being there” while in a simulated reality (Riva et al., 2011; Waterworth et al., 2015). Second, VR can be used to generate complex vast stimuli, which can target specific facets of awe (Shiota et al., 2007). Third, VR can be used to conveniently track behavioral and physiological responses, allowing an integrated assessment of the emotional experience. We conclude by discussing the potential challenges related to the implementation of the proposed strategy.

## Research on Awe

Awe is a complex emotion arising from the perception of and need to accommodate vastness (Keltner and Haidt, 2003; Shiota et al., 2007), and which is able to impact on several psychological systems. Thus far, awe has begun to accrue a small research literature investigating its aspects. For example, it has been shown that the experience of awe can foster prosocial attitudes as measured by the percentage of money participants allocate to others in distribution tasks (Prade and Saroglou, 2016). Awe can also influence beliefs. Participants in one study were more likely to endorse spiritual-type beliefs after feeling awe (Van Cappellen and Saroglou, 2012). Awe also influences well-being and physical health (Krause and Hayward, 2015; Stellar et al., 2015). Furthermore, as an epistemological emotion (Keltner and Shiota, 2003; Shiota et al., 2007), awe can change people’s general perspectives toward the world and themselves (Schneider, 2009). Awe also has been shown to effect the detection of agency (Valdesolo and Graham, 2014). One study has found that the experience of awe changes one’s sense of time (Rudd et al., 2012). Finally, other studies have explored awe’s phenomenological dimensions, identifying themes such as “fear,” “connectedness,” “existential awareness,” and “numinous” (Bonner and Friedman, 2011; Gallagher et al., 2015).

Most empirical studies have explicitly referred to the theoretical model developed by Keltner and Haidt (2003). The flexibility of valence in awe is somewhat unusual for an emotion, though there are other examples that follow this pattern, such as nostalgia (Wildschut et al., 2006). Since conventional models of emotions typically do not admit this degree of complexity (Trnka et al., 2016), Keltner and Haidt (2003) suggested the existence of different emotional themes (i.e., threat, beauty, virtue, power, and uncanny) that “flavor” awe (Keltner and Haidt, 2003; p. 304), creating a kind of family of awe-related states. For example, awe arising from threatening stimuli can be a source of negative valence, as they write, “danger causes an experience of awe to be flavored by feelings of fear” (Keltner and Haidt, 2003; p. 304). Finally, supernatural or sublime stimuli, such as an experience of God, can flavor awe with a negative or positive component, such as fear or love (Keltner and Haidt, 2003).

Qualitative research has also been conducted on awe. This research mainly relies on interviews investigating phenomenological aspects of awe. These studies have generally found evidence for profound, intense, self-altering, and occasionally transformative capacities of awe (Bonner and Friedman, 2011; Gallagher et al., 2015).

Despite these complexities of awe, experimental research has tended to focus on only a few parts of this theoretical model. Indeed, the negative side of awe has been explored by few researches (e.g., Shiota et al., 2007; Piff et al., 2015). Further, the need for accommodation component has only rarely been investigated, maybe due to difficulties in its operationalization. Indeed, Keltner and Haidt themselves struggled to provide an example of the need for accommodation dimension, as suggested by Sundararajan (2002). While few studies have explored this dimension experimentally a few exploratory studies offer promising new directions. For instance, Schurtz et al. (2012) investigated need for accommodation by asking participants to remember a moment of awe in their lives and then measuring different aspects of awe. They found that the need for accommodation dimension did not predict the occurrence of awe among participants. Other studies investigated the role of personality factors related to the need for accommodation, showing the predictive role of Openness to Experience on the tendency to experience awe (Silvia et al., 2015), the inverse relationship between dispositional awe and the Need for Cognitive Closure (Shiota et al., 2007), and the positive relationship between the amount of awe experimentally induced and a reduced ability to deal with uncertainty (Valdesolo and Graham, 2014).

Negative awe experiences have been shown to be less frequent than positive ones (Shiota et al., 2007; Bonner and Friedman, 2011), but this does not justify the reduced research attention to the negative dimension of awe (Bonner and Friedman, 2011). On the contrary, recently, some positive psychology researchers explicitly characterized awe as a Positive Emotion (Griskevicius et al., 2010; Shiota et al., 2011; Van Cappellen and Saroglou, 2012; Campos et al., 2013; Prade and Saroglou, 2016). Most experimental studies have considered awe according to this characterization, which has proliferated in recent years (Bonner and Friedman, 2011). The negative dimension of awe needs further elucidation.

In sum, while awe is beginning to accrue a small research literature, certain aspects of awe detailed in the theoretical literature have been underexplored. In particular, research has tended to: (i) emphasize vastness rather than the need for accommodation; (ii) focus on positive rather than negative awe experiences; and (iii) rely on relatively low-intensity awe experiences. While the first two issues need to be addressed at theoretical level, we contend that VR could provide an effective solution to address the third challenge, that is, how to induce more intense and ecologically valid awe experiences. Silvia et al. (2015) argued that intense experiences of awe have not been induced in laboratory settings, writing that awe experiences seem “too rare and eccentric to be captured in the lab” (p. 382). These authors called for an integration of different methods and disciplines that have studied awe, in order to “place the findings from low-intensity and small-scale lab research in context” (p.382).

## Using Virtual Reality to Study Awe

A recent comprehensive work on existing literature on VR and personal change, reported the effective use of VR in several domains, such as addictions, anxiety disorders, stress related disorders, depression, eating disorders, and mood induction (Riva et al., 2016b). Regarding the latter aspect, virtual experiences can really influence our inner world, such as the emotional one (Dobricki and Pauli, 2016). In details, VR has been used to to elicit both positive and negative emotions. For example, in the study of Riva et al. (2007) participants were immersed in three virtual parks designed to induce different emotional states (i.e., anxiety, relaxation and neutral). Each video was displayed through a head-mounted device able to track head movements. Parks shared the same basic structural components (e.g., trees, lamps, band stand) but they differed regarding some other environmental elements such as music, light, and textures. For example, the virtual park intended to evoke sadness was gray and cloudy, users were alone, and sadness-eliciting music was selected (i.e., “Adagio for Strings-Choral” by Samuel Barber). The neutral virtual park, in contrast, had different music (i.e., “Nothing Spectacular” by Michael Lindh). For the validation phase of each park all participants listened to short history, which was different according to the emotional experimental condition (Baños et al., 2004). Findings indicated that virtual stimuli were able to induce the expected emotional states. Recently, this procedure was replicated and further extended by Felnhofer et al. (2015) who used five different virtual park scenarios to elicit specific affective states. The researchers created virtual park scenarios for joy, anger, boredom, anxiety, and sadness, which shared the same layout (i.e., non-playing virtual characters; trees; rubbish bins; park benches; a pond). As these series of studies demonstrate, VR allows for the targeted manipulation of certain elements of the environment in order to elicit target emotions.

A first attempt to use virtual simulation to elicit awe was made by Reinerman-Jones et al. (2013). These authors analyzed participants’ experiences of viewing the earth from a deep space perspective – the so called the “Overview Effect” (White, 1987; Yaden et al., 2016) – using a qualitative analysis. In another study, Gallagher et al. (2015) induced the Overview Effect using a mixed reality test environment, in which participants watched four simulations of the earth or deep space as if they were astronauts on the International Space Station. Results of this experiment showed that VR, along with traditional methods, such as autobiographical recall, videos, and narratives can be used together to induce awe experiences. This provides an initial, exploratory study, which we believe can be usefully built upon. In particular, we argue that VR presents the following assets for the investigation of awe.

### VR Enhances Presence and Ecological Validity

Virtual reality is a technological system that combines sensorial displays (i.e., visual, auditory) with tracking devices that sense the movements of the individual and report the collected data to the visualization system, which updates the scene in real time. VR allows researchers to create situations closer to equivalent real ones, thanks to the pictorial realism and the high resolution provided (Coelho et al., 2006; Parsons, 2015). Pictorial realism is a function of visual depth illusion, which is better provided by 3D virtual environments than by 2D videos on flat computer screens. Furthermore, VR supports more intense involvement by users. The sensorial richness provided by VR describes the number of sensorial channels involved during virtual experiences (Steuer, 1992; Coelho et al., 2006). Also, the isolation offered by some VR devices can provide an extremely vivid sense of presence in the virtual environment (Riva and Mantovani, 2012, 2014; Waterworth et al., 2015). In practical terms, presence allows researchers to access more reflexive responses from participants, as responses are closer to those produced by real-world circumstances, as opposed to those produced by watching a video on a screen. Finally, Gorini et al. (2011) showed that providing a narrative with VR involving long-term goals and an engaging stroyline promoted a fuller sense of immersion in virtual environments. This narrative component is another relevant feature of VR to elicit stronger and more meaningful experiences (Triberti and Riva, 2015).

### VR allows the Generation of Complex and Vast Stimuli

As mentioned previously, complex and vast stimuli enhance awe (Keltner and Haidt, 2003; Shiota et al., 2007). VR has the potential to generate such stimuli by delivering embodied experiences that overcome our sense of physics and challenge our assumptions about the world (Riva, 2016). From a psychological standpoint, VR can be described as an “embodied technology” that is able to manipulate the feeling of being and acting in the world (Riva, 2009; Riva et al., 2014). According to the Embodiment Perspective on cognition, cognitive components of emotions are not separated from the body, since cognition is derived from the close relationship among body, environment and brain (Varela et al., 1991; Thelen et al., 2001; Beer, 2003). VR, as an embodied medium, allows to manipulate the “cognitive being’s world” (Colombetti and Thompson, 2008; p.56), by providing different perceptual cues to the users and by inviting the user to build a specific representation of the situation in which she/he is involved. For example, users can experience the virtual environment as if it was “his/her surrounding world” (*augmented embodiment*) or can experience a synthetic avatar (user’s virtual representation) as if it was “his/her own body” (*synthetic embodiment*).

For example, VR can be used to alter the perceptual boundaries of the body through the “incarnation” of the subject in the virtual space (Riva and Mantovani, 2012, 2014). For instance, participants could experience *ad hoc* “time travels” (Friedman et al., 2014). In this case, participants were involved in a moral dilemma in which they experienced a sequence of events (in this case, the opportunity to stop a gunman from killing others) in VR from a first person perspective. In the time travel condition, people came back to the beginning, and saw their past selves’ with the possibility to change events. This illusion is based on altering participants’ accustomed frames of temporal reference, resulting in an impact on individuals’ moral judgment on themselves, as well as influencing their actions and choices to solve the moral dilemma.

A further example is related to the use of VR for the manipulation of *bodily self-consciousness*, that is, the replacement of the bodily self-consciousness with a synthetic one (Riva, 2016). In this approach, VR is used for creating synthetic avatar (user’s virtual representation) experienced by the user as if it were his/her own body. To achieve synthetic embodiment, a space-temporal correspondence between the multisensory signals and sensory feedback experienced by the user, and the visual data related to the avatar, are required. For instance, Serino et al. (2016) invited individuals to embody a skinny virtual body, and found a decreased estimation of difference between their virtual avatar and their actual body. In other words, people thought they were thinner after having a virtual experience of being thin.

Researchers can create experimental VR paradigms from a number of unusual first-person perspectives (Rosenberg et al., 2013). In one experiment conducted by Osimo et al. (2015), participants switched between a virtual body closely resembling themselves where they described a personal problem, and a virtual body representing Dr Sigmund Freud, from which they offered themselves counseling. These researchers report that when the counselor resembled Freud, participants’ mood improved compared to when the virtual counselor was a self-representation. In this regard, VR could be considered as a source of “epistemic expansion” in the sense that it provides the possibility of experiencing events that would be otherwise inaccessible (Gaggioli, 2016).

### VR as an Integrated Measurement Tool

Virtual reality can provide two main ways of tracking the users’ emotional awe responses, at both behavioral and physiological level. Using VR, it is possible to measure users’ behavior in real time during the experience, using motion-tracking device systems. These devices allow tracking of head-movements, upper-limb and hands and facial-movements, as well as measuring posture. This information could be used to analyze, for example, non-verbal displays of awe. Further, VR offers the potential to monitor psychophysiological correlates of awe, for example measuring Skin Conductance, Heart Rate, Skin Temperature, and Respiration, while the participant is exposed to awe inducing stimuli, delivered through the immersive medium. More importantly, VR allows integrating these behavioral and psychophysiological measurement with the self-reported descriptions of the experience in an ecological but controlled setting (Wiederhold and Rizzo, 2005; Parsons, 2015: Fusaro et al., 2016). For example, dynamic stimuli could be conveyed to participants trough VR immersive displays, and their psychophysiological reactions, as well as their facial movements, or postural ones, could be concurrently assessed and integrated to build a more comprehensive model of the emotional experience.

## Possible Issues Concerning The Use Of Virtual Reality In Awe Research

While the use of VR to elicit awe can be promising, there are several warnings and guidelines that researchers should keep in mind. The aspects of VR that effectively raise the intensity of awe experiences also increase the risk of a negative reaction in participants.

First, as a note to interested researchers, some VR set-ups can be needlessly expensive. Reinerman-Jones et al. (2013) used a mixed reality simulation as a new tool to investigate the phenomenological emergent features of awe experience. Indeed, they were also able to grasp an element of surprise, closer to the need for accommodation component of awe as well as a negatively valenced awe experience by creating a highly ecological experience of awe (Gallagher, 2013). This study provides support for our argument (though they did not quantitatively measure awe), nevertheless, they adopted a very expensive solution. However, new mobile affordable inexpensive VR solutions are becoming more widely available, as Samsung Gear VR, Google Cardboard, and HTC Vive. Second, VR can also cause motion sickness. From the user side, the eventual mismatch between visual and vestibular information (Reason and Brand, 1975), can be a source of complaint, and researchers should be aware of it in order to intervene immediately and adequately. The likelihood of suffering from this kind of disorientation increases if people are requested to “walk” into the virtual environment or to stand up for long periods of time. Therefore, VR should be used for relatively short periods of time and include a debriefing session, especially if negative emotions were a target.

Lastly, participants should receive adequate informed consent information, particularly if an emotionally intense virtual environment will be used. For example, some virtual environments intended to induce a negative awe experience may become terrifying for some participants, prompting an adverse reaction, in some cases maybe requiring debriefing or even a referral to a psychotherapist. It is imperative that participants are aware of the kind and level of intensity of the experience they will undergo.

## Conclusion

Awe is a profound and complex emotion, which can change peoples’ lives. It can be considered as the core of transformative and enduring change (Gaggioli, 2016), even though this potential has not been fully explored in experimental research. Indeed, experimental research on awe has constructively manipulated awe, but awe inductions in laboratory settings have tended to be quite subtle, producing low intensity instance of awe. We proposed VR as one possible solution to disclose awe potential. Several studies support the potential of VR as a transformative technology, able to induce a personal change (Ferrer-Garcia et al., 2013; Garrett et al., 2014; Den Brok and Sterkenburg, 2015; Riva et al., 2016b). VR allows simulating both our outer and inner world (Riva et al., 2016b), by enhancing individuals’ focus on themselves, thus recreating both external and internal correlates of our experience, but preserving highest levels of experimental control and ecological validity. Furthermore, with VR it is possible to reproduce complex and vast stimuli at the base of awe emergence. Moreover, VR allows for a multi-level assessment of awe that could be useful to catch more awe nuances. Finally, if safety and effectiveness recommendations regarding this experimental tool are respected, we believe VR can play an important role in the study of awe as well as other emotions.

## Author Contributions

Authors contributed according to their competences and interests. AC and AG conceived the main idea of the article. AC wrote the first draft of the manuscript, while GR and DY contributed to the final writing of the manuscript by giving suggestions regarding the issues related to the rhetoric and to the literature. AG supervised the entire work. All authors contributed to the manuscript, read, and approved the final version.

## Conflict of Interest Statement

The authors declare that the research was conducted in the absence of any commercial or financial relationships that could be construed as a potential conflict of interest. The reviewer OM and the handling Editor declared their shared affiliation, and the handling Editor states that the process nevertheless met the standards of a fair and objective review.
